# Parametric analysis of transcatheter aortic valve replacement in transcatheter aortic valve replacement: evaluation of coronary flow obstruction

**DOI:** 10.3389/fbioe.2023.1267986

**Published:** 2023-10-11

**Authors:** Roberta Scuoppo, Stefano Cannata, Giovanni Gentile, Caterina Gandolfo, Salvatore Pasta

**Affiliations:** ^1^ Department of Engineering, Università Degli Studi di Palermo, Palermo, Italy; ^2^ Department for the Treatment and Study of Cardiothoracic Diseases and Cardiothoracic Transplantation, IRCCS Istituto Mediterraneo per i Trapianti e Terapie ad Alta Specializzazione (ISMETT), Palermo, Italy; ^3^ Radiology Unit, Department of Diagnostic and Therapeutic Services, IRCCS-ISMETT, Palermo, Italy; ^4^ Department of Research, IRCCS-ISMETT, Palermo, Italy

**Keywords:** transcatheter aortic valve replacement, finite element analysis, computational fluid dynamic, transcatheter heart valve, TAVR-in-TAVR

## Abstract

Transcatheter aortic valve replacement (TAVR) is increasingly being considered for use in younger patients having longer life expectancy than those who were initially treated. The TAVR-in-TAVR procedure represents an appealing strategy to treat failed transcatheter heart valves (THV) likely occurring in young patients. However, the permanent displacement of first THV can potentially compromise the coronary access and ultimately inhibit the blood flow circulation. The objective of this study was to use finite-element analysis (FEA) to quantify coronary flow in a patient who underwent TAVR-in-TAVR. A parametric investigation was carried out to determine the impact of both the implantation depth and device size on coronary flow for several deployment configurations. The FEAs consisted of first delivering the SAPIEN 3 Ultra THV and then positioning the Evolut PRO device. Findings indicates that high implantation depth and device undersize of the second THV could significantly reduce coronary flow to 20% of its estimated level before TAVR. Additionally, a positive correlation was observed between coronary flow and the valve-to-coronary distance (R = 0.86 and *p* = 0.032 for the left coronary artery, and R = 0.93 and *p* = 0.014 for the right coronary artery). This study demonstrated that computational modeling can provide valuable insights to improve the pre-procedural planning of TAVR-in-TAVR.

## 1 Introduction

Transcatheter aortic valve replacement (TAVR) has now become the standard of care for elderly patients with aortic stenosis, as compared to conventional open-chest surgery (Leon et al., 2010). Ongoing clinical trials are currently investigating the feasibility and safety of TAVR in young and bicuspid patients, who were previously excluded from earlier trials (De Backer et al., 2020; Forrestal et al., 2020; Nai Fovino et al., 2020). This is important, as young patients have a longer life expectancy than those who were initially treated with TAVR. However, even if there are no concerns about the durability of transcatheter heart valves (THV), a substantial proportion of contemporary TAVR patients is expected to live long enough to experience the degeneration or failure of the implanted device. The long-term durability of THVs remains a major challenge, as the gradual deterioration of biological valve leaflets due to calcification and thrombosis can occur after implantation (Scardulla et al., 2017; Cosentino et al., 2019). Indeed, valve thrombosis is usually occurring after 3-year with an incidence of 9.3% in TAVR patients (Bosi et al., 2018).

In this setting, the deployment of a new THV in the failed one (namely, TAVR-in-TAVR) is an attractive therapeutic option to further extend the life expectancy of TAVR patients. Although TAVR-in-TAVR is clinically feasible, the new THV permanently displaces the valve leaflets of the first device towards the metallic stent frame by generating a tube graft that extends from the annulus to the sinus of Valsalva or above the sino-tubular junction. This not only impairs coronary access but also compromises coronary flow. Using computed tomography (CT), Forrestal and colleagues (Forrestal et al., 2020) demonstrated that the self-expanding Evolut Pro device can lead to a risk of coronary obstruction in up to one in four patients, and future coronary access may not be possible or may be extremely challenging in up to four in five patients after TAVR-in-TAVR. To avoid coronary complications, it is possible to identify a risk plane below which the first valve frame should not be crossed after TAVR-in-TAVR (Nai Fovino et al., 2020). However, anatomic constraints and device characteristics remain the main challenges in the pre-operative planning of TAVR-in-TAVR. Indeed, the distance from the metallic stent frame of the second THV to the coronary ostia or the extension of the THV skirt above coronary arteries can negatively affect the flow circulating in the arteries. Only a few imaging studies have attempted to predict the likelihood of coronary obstruction based on morphologic parameters and anatomic dimensions, but they are not considering the device-host interaction (De Backer et al., 2020; Forrestal et al., 2020; Nai Fovino et al., 2020).

With this in mind, we conducted a study to determine the flow of blood circulating in coronary arteries under different implantation configurations and device sizes using patient-specific computational analysis of TAVR-in-TAVR. Specifically, we investigated a patient with a degenerated SAPIEN 3 Ultra THV that was treated with the self-expanding Evolut Pro during a second treatment. By simulating the deployment of each device and performing smoothed-particle hydrodynamic (SPH) analysis, we not only evaluated the feasibility of TAVR-in-TAVR, but also gained a better understanding of how hemodynamic performance varies with different deployment configurations.

## 2 Methods

We investigated the case of a 68-years-old gentleman with severe aortic stenosis, which was treated with a 23-mm SAPIEN 3 Ultra THV. Baseline patient characteristics were: heartbeat of 65 bpm, systolic blood pressure of 113 mmHg, diastolic pressure of 65 mmHg, peak gradient of the stenotic valve of 60 mmHg, transaortic flow jet of 3.9 m/s, aortic valve annulus of 22.8 × 23.0 mm and calcium volume of 1,387.4 mm^3^. After 3 years from TAVR, early device degeneration characterized by valve thrombosis and leaflets thickening was observed during echocardiography. Specifically, hypo-attenuated leaflet thickening of SAPIEN 3 Ultra THV with reduced leaflet motion was observed. Based on the surgical risk and patient clinical history, the Heart Team decided to implant a 29-mm Evolut PRO device through TAVR-in-TAVR to treat the degenerated bioprosthesis. A strict ECG-gated CT imaging was performed to evaluate the stenotic aortic valve before and after each device implantation. Therefore, a total of three ECG-gated CT studies were conducted for this patient:

• The first was to evaluate the aortic valve annulus of the diseased valve (i.e., pre-TAVR).
• The second aimed to assess the functionality of the initially implanted bioprosthesis (i.e., TAVR).
• The third was conducted to evaluate the deployment of the second bioprosthesis (i.e., TAVR-in-TAVR).

ECG-gated CT images of the patient prior to TAVR were processed in Mimics (v.21, Materialize, Belgium) to reconstruct the geometry of the aortic root. This involved semi-automatic thresholding of contrast-enhanced images, followed by manual editing and smoothing (Scardulla et al., 2017). Calcific plaques were identified by creating a separate mask and applying automatic thresholding to the bright calcium image. Stenotic valve leaflets were modeled using anatomic measurements and 3rd-order NURBS curves in Rhinoceros software (Rhinoceros v.7, McNeel and associates, United States). Specifically, leaflet free edges were manually segmented by spline curves in the axial plane after multiplanar reformations of diastolic images. The leaflet-to-sinus attachments were identified by spline curves generated on the aortic root surface. Each leaflet was modeled using a curve that was constrained on the curves of both the leaflet-free-edges and leaflet-to-sinus, with a control point at the midpoint to model the curvature of valve leaflets. The leaflet-to-sinus curves were projected onto the aortic root surface, and then the final shape of the native valve leaflets was developed using a multi-patch surface network.

Each anatomic part was meshed using ICEM meshing software (v2021, ANSYS Inc., United States) after convergence analysis. We used the stress parameter (i.e., the Mises stress) to calculate the discretization errors and estimates were kept <5%. Thus, the patient-specific model was meshed with unstructured triangular shell elements for the aortic root surface (with a size of 0.6 mm) and tetrahedral solid elements for calcifications (with a size of 0.5 mm). Native valve leaflets were initially meshed with triangular shell elements and grid size of 0.12 mm, which were then protruded to generate a solid part with four layers of elements along the thickness direction. Table 1 summarizes the number of mesh elements for each anatomic component.

**TABLE 1:** Material parameters used for patient-specific models and bioprosthesis; E = Young modulus; ν = Poisson coefficient; C10 = material constant; D1 = incompressibility factor; *σ*_y_ = yield stress; *σ*_ult_ = ultimate tensile stress; *ε*_p_ = plastic strain; μ = viscosity; D = density.

	E (MPa)	ν	C10 (MPa)	D1 (MPa^-1^)	σ_y_ (MPa)	σ_ult_ (MPa)	ε_p_	μ (Pa s)	D (kg/m^3^)	Element number (thousand)
Aorta			1.05	0.048						109.1
Native Leaflet			2.29	0.022						9.6
Calcification	400	0.47							2,000	62.2
S3 Ultra	233e+3	0.35			414	930	0.45		8,000	84.6
S3-Skirt	55	0.49			6.6	6.6	0.6		1,060	23.1
Balloon									1,060	82.3
Evolut Pro									8,000	317.4
Evolut-Skirt	55	0.49			6.6	6.6	0.6		1,060	63.1
Crimper and Sleeve									1,060	6.9
Fluid								3.7 × 10^−3^	1,060	512.1

To calibrate the biomechanical response of the aortic root and valve leaflets, we developed an inverse method using the ECG-gated CT images collected before TAVR as done previously (Cosentino et al., 2019). The approach consisted of the simultaneous minimization of two cost functions, which represented the difference between model predictions and CT measurements of aortic wall strain and orifice area at the systolic phase. The diastolic-to-systolic aortic wall strain of 4.3% was measured as the changes in aortic diameters between systole and diastole divided by the diastolic diameter, while the orifice area of stenotic valve leaflets was 104.5 mm^2^ at CT scan. We assumed a Neo-Hookean material model for the material behavior of both the aortic root and native valve leaflets. Calcifications were embedded in the native valve leaflets and had a linear-elastic material behavior (E = 400 MPa and *n* = 0.47) (Bosi et al., 2018). The inverse approach used a quadratic regression model to link the unknown input variables (i.e., the Neo-Hookean material parameter) to the output variables (i.e., the aortic wall strain or the orifice area). By utilizing the data from CT imaging, an optimal set of material properties for the aortic wall and valve leaflets was determined. This approach successfully minimized the difference in circumferential strain between diastole and systole, achieving a relative error of less than 5% when compared to CT measurements. Table 1 summarizes the material properties for the patient-specific model.

### 2.1 TAVR-in-TAVR simulation

The structural heart intervention was simulated using finite-element analysis (FEA) with Abaqus/Explicit software (v2021hf7, Dassault Systèmes, United States). To model the dynamic phenomenon, we used a quasi-static FEA approach that ensured the kinetic to internal energy ratio remained below 10%. This was achieved through a mass scaling technique using a stable time increment and thus reducing the computational cost. In addition, a penalty contact algorithm with a friction coefficient of zero was used to enable contact during simulations.

Bioprosthesis models were meshed with structured hexahedral elements, as reported in previous studies by our group (see Table 1) (Pasta et al., 2020a; Pasta et al., 2021). Stainless steel with bilinear elasto-plastic material and superelastic Nitinol alloy (14 constants user material (Morganti et al., 2014)) were employed to model the SAPIEN 3 Ultra and Evolut PRO, respectively. The device sealing skirt was considered closing the cell geometries of each stent frame with several surfaces modelled at mid-thickness of each device frame. For the SAPIEN 3 Ultra, all cell-structs of the THV frame were closed to mimic the displaced shape of valve leaflets as seen in TAVR-in-TAVR. This surface, which represents the device skirt and leaflets, was modelled at the crimped phase of SAPIEN 3 Ultra deployment in the human model. For the Evolut PRO, the skirt was included at end of TAVR-in-TAVR simulation by mapping the skirt surface on the stent frame and resolving potential overclosure by strain-free adjustments analysis in Abaqus\Explicit. Skirts were meshed with triangular membrane elements with a thickness of 0.1 mm and were then connected to the device frame using tie contact conditions.

Crimping of SAPIEN 3 under frictionless contact conditions was carried out using a cylindrical surface gradually moved along the radial direction from the initial device diameter of 23 mm to the final diameter of 6 mm at the device crimped stage. Then, the SAPIEN 3 Ultra device was placed in the patient-specific model with an implantation depth of 5 mm whilst a restart analysis was considered to account for the stress state resulting from the crimping numerical analysis. After crimping, an elastic recoil of SAPIEN 3 Ultra was observed. For the sake of simplicity, expansion of SAPIEN 3 Ultra THV was performed by radially displacing a cylindrical surface representing the wall of the expanding balloon. Expansion was obtained by enlarging the balloon surface upon the device nominal diameter of 23 mm. This can be considered a valid approach since angiography usually highlights negligible axis rotation and translation during device expansion.

After deploying the SAPIEN 3 Ultra, the Evolut Pro was positioned in the post-TAVR model with an implantation depth of 9 mm and was then crimped with an approach similar to that of first device. By pulling the sleeve towards the distal ascending aorta and releasing the device, the Evolut Pro stent was gradually deployed into the first device to mimic the TAVR-in-TAVR.

A parametric analysis of the TAVR-in-TAVR procedure was carried out by varying the implantation depth and device size with respect to the baseline model. The following scenarios were therefore explored:

• 5-mm implantation depth for the 23-mm SAPIEN 3 Ultra and 9-mm implantation depth for the 29-mm Evolut Pro (i.e., the S3-in-Ev)—reference model as clinically done by the Heart Team.
• 5-mm implantation depth for the 23-mm SAPIEN 3 Ultra and 16-mm implantation depth for 29-mm Evolut Pro (i.e., the S3-in-EvL)—low TAVR-in-TAVR implantation
• 2-mm implantation depth for the 23-mm SAPIEN 3 Ultra and 9-mm implantation depth for the 29-mm Evolut Pro (i.e., the S3H-in-Ev)—high TAVR implantation
• 5-mm implantation depth for the 23-mm SAPIEN 3 Ultra and 9-mm implantation depth for the 26-mm Evolut Pro (i.e., the S3-in-Ev26)—device undersizing of TAVR-in-TAVR

### 2.2 Fluid-solid interaction analysis

**FIGURE 1:**
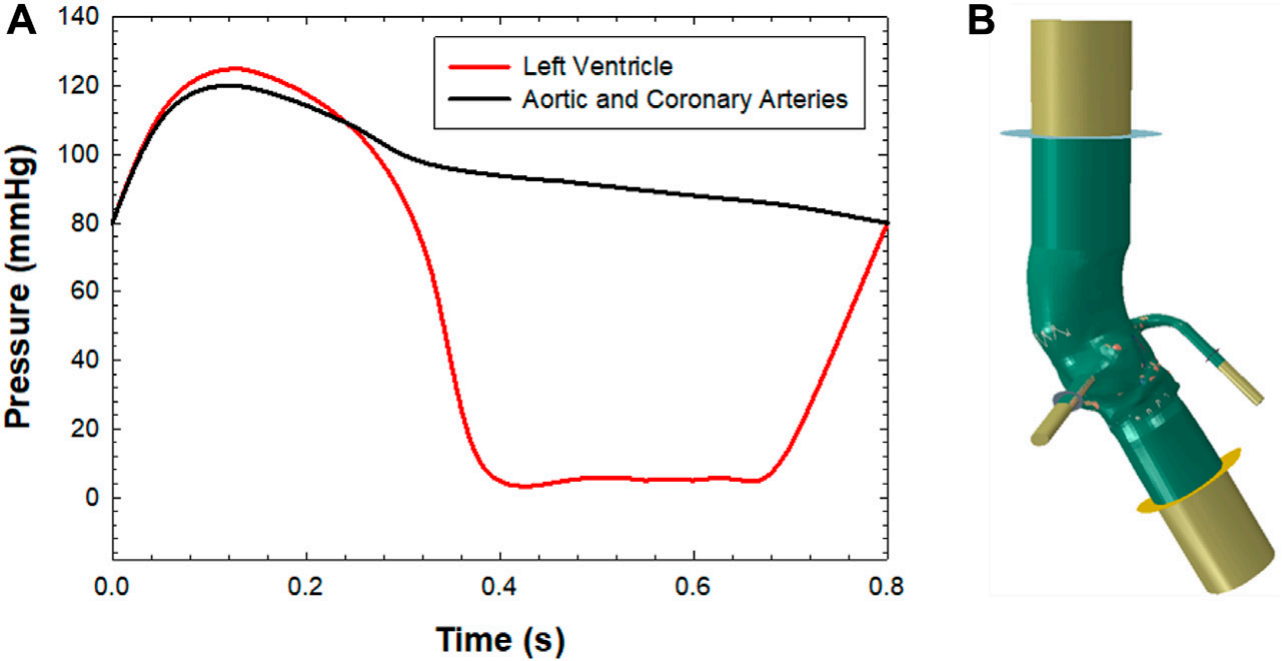
**(A)** Pressure boundary conditions applied to each plate for simulating fluid motion and **(B)** SPH model with the aortic wall, extension and rigid plates for applying the boundary conditions.

After the TAVR-in-TAVR simulation, the smoothed-particle hydrodynamics (SPH) modeling approach was used to quantify flow circulating in coronary arteries. The SPH approach, adopted in previous studies (Mao et al., 2016; Caballero et al., 2017; Caballero et al., 2020), is useful for modeling extreme deformation and complex contact problems in the kinematics of aortic and mitral valves. The blood was modelled as a Newtonian fluid characterized by a density of 1060kg/m3 and viscosity of 0.0035 Pa s, using the pressure-density relation governed by the linear Hugoniot equation of state (artificial sound speed of c0 = 145 m/s). For particle discretization, the fluid domain confined by the aortic lumen was meshed with unstructured tetrahedral elements for all TAVR-in-TAVR configurations (see Table 1). Flow motion was generated by pressure boundary conditions exerted on the fluid volume by several rigid plates located at the proximal and distal ends of the aortic wall and coronary arteries, as previously reported by our group (Figure 1) (Caballero et al., 2020). This allowed the development of a transmural pressure gradient across the device valve leaflets according to physiological pressure waveforms generated by the beating heart. Tie contact conditions were developed between the fluid and rigid plates to avoid separation among parts. This allowed the transfer of the pressure applied to each rigid plate in fluid motion. Using a predefined field in Abaqus, an initial fluid pressure of 80 mmHg was set for the SPH particle. For coronary arteries, the rigid plates were loaded with a pressure waveform identical to that of the distal aortic wall. However, we did not consider the systolic increase in hydraulic resistance of the coronary circulation. The proximal end of the aortic wall and coronary arteries was extended 5-fold the annulus diameter to reduce transient effect during the simulated cardiac beat (time step of 0.8 s). The cardiac output was 5 L/min assuming a systolic time of 0.3 s. Two cardiac cycles were simulated, and the second was used for data analysis (each SPH simulation lasted 21.6 h). The general contact algorithm in Abaqus/Explicit enabled the FSI interaction between fluid particles and device valve leaflets. For post-processing, particle flow data were mapped on a solid tetrahedral element mesh of the fluid domain to analyze flow velocity in contour map instead of discrete particle point collection.

### 2.3 Data analysis

To compare CT images of TAVR-in-TAVR with FEA predictions, the device expansion was computed using the nominal prosthesis size as a follow (observed THV external area/ device area nominal size) × 100. Specifically, measurements were taken at three cross-sectional level including the inflow, mid and outflow of TAVR-in-TAVR. The valve-to-coronary (VTC) distance was measured in the short-axis view after multiplanar reformations as the length from the metallic device frame to the ostia of left coronary artery (LCA) and right coronary artery (RCA). The valve-to-coronary (VTC) distance was measured in the short-axis view as the length from the metallic device frame to the ostia of left coronary artery (LCA) and right coronary artery (RCA). The coronary flow percent was computed as the ratio of the coronary flow for each TAVR-in-TAVR scenario divided by the flow of the model prior to TAVR-in-TAVR. The linear relationship between coronary flow percent and VTC distance was investigated by Pearson correlation.

## 3 Results

**FIGURE 2:**
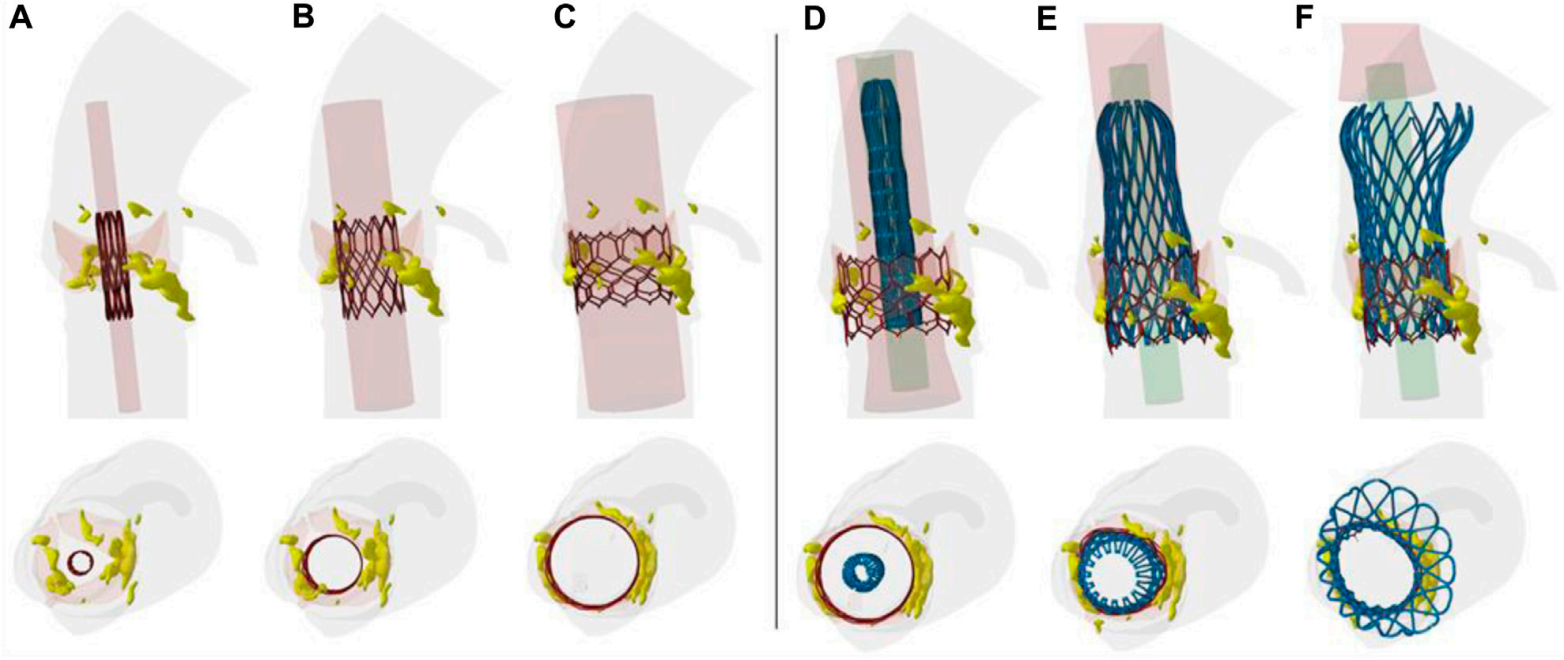
Different steps of TAVR-in-TAVR simulation; the deployment from the crimped THV to **(A–C)** ex-panded SAPIEN 3 Ultra and from the Evolut PRO to the **(C–F)** final TAVR-in-TAVR is shown.

Several steps of the deployment of both SAPIEN 3 Ultra and Evolut Pro THVs are shown for the axial and sagittal views of the patient anatomy (Figure 2). After crimping of first device, the cylindrical surface allowed the delivery of the SAPIEN 3 Ultra. The latter permanently displaced the native valve leaflets and calcifications. Later, the self-expanding devices was gradually released to further expand the vessel and replace the degenerated THV.

**FIGURE 3:**
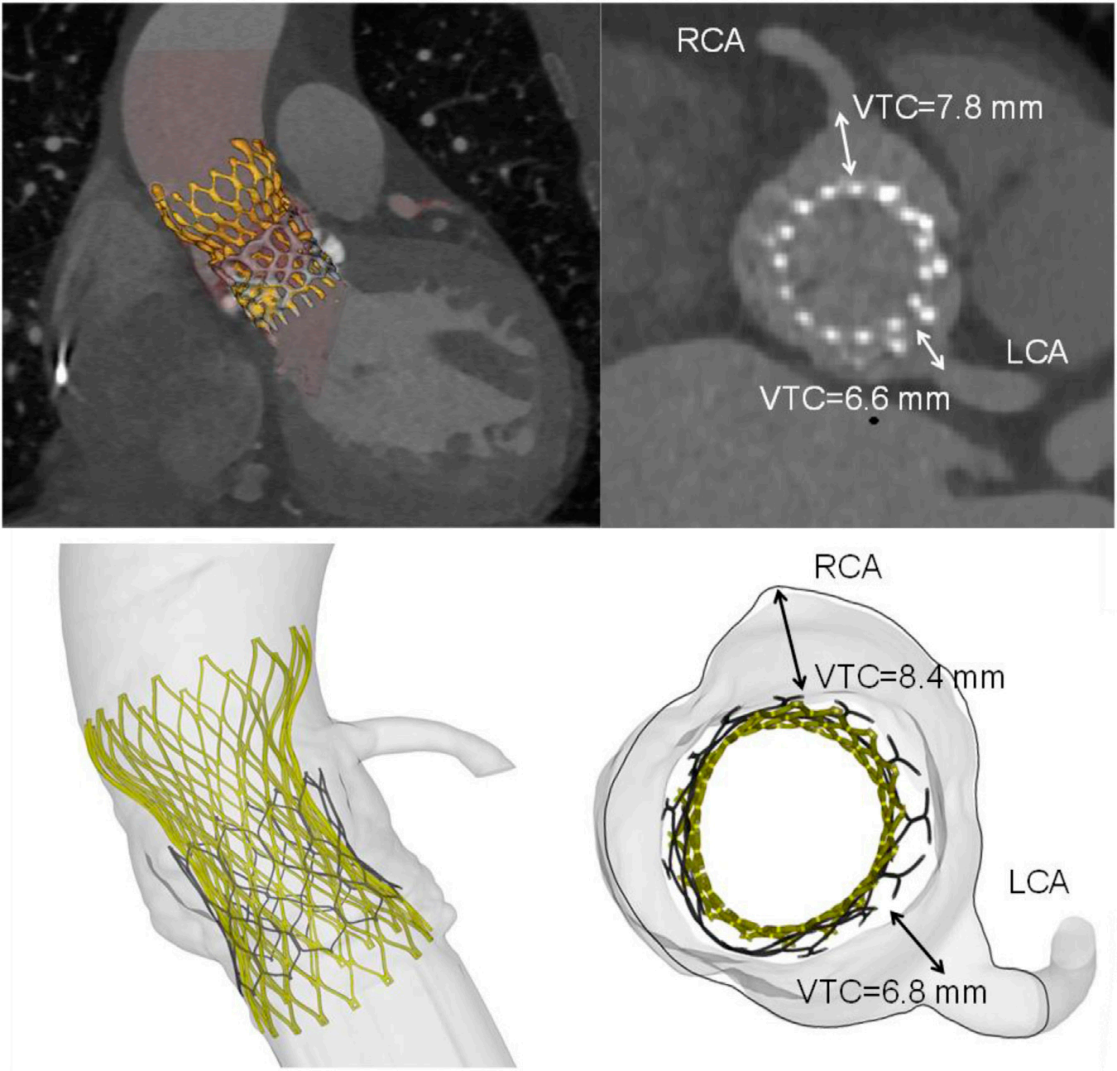
Volumetric rendering of TAVR-in-TAVR and axial views of implanted device (top row) as compared to simulation results (bottom row). Insets shows both CT- and FEA-based VTC measurements. The deployed Evolut device had an oval shape with minor and major diameters of 23.4 mm and 26.3 mm, respectively.

**FIGURE 4:**
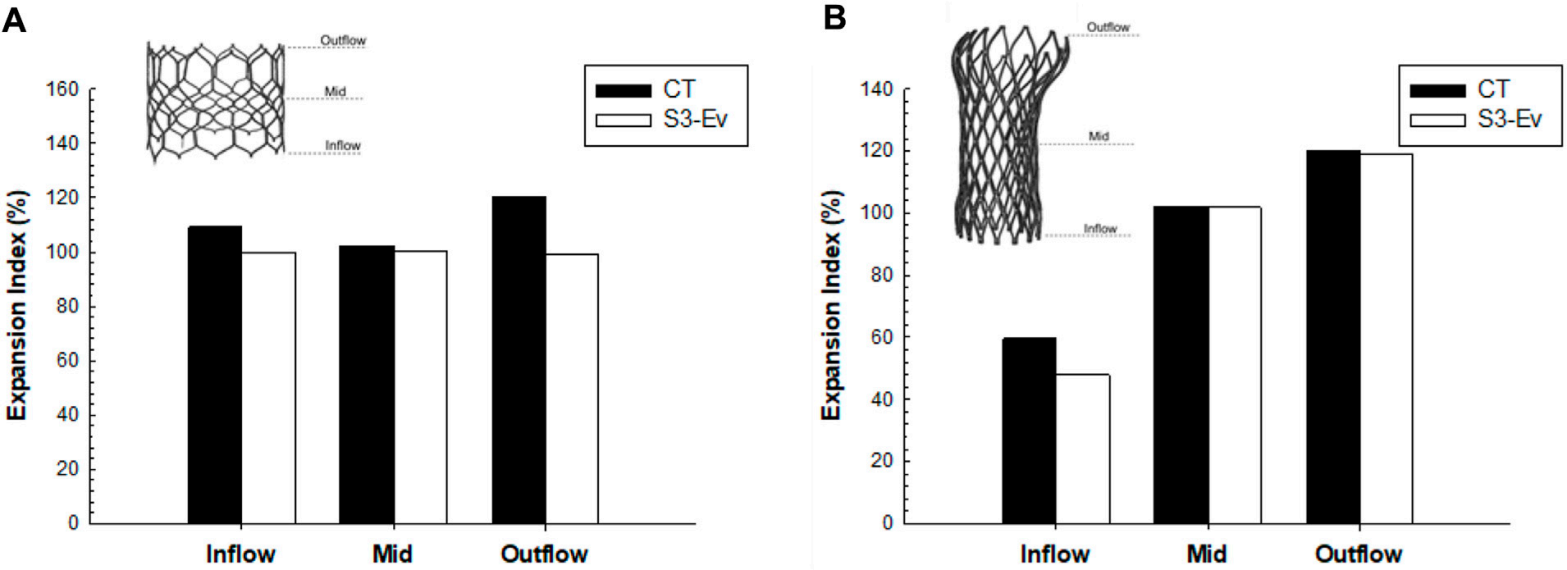
Comparison of expansion index at different anatomic levels of each THV between CT measures and FEA predictions for **(A)** SAPIEN 3 Ultra and **(B)** Evolut Pro for the baseline model.

**FIGURE 5:**
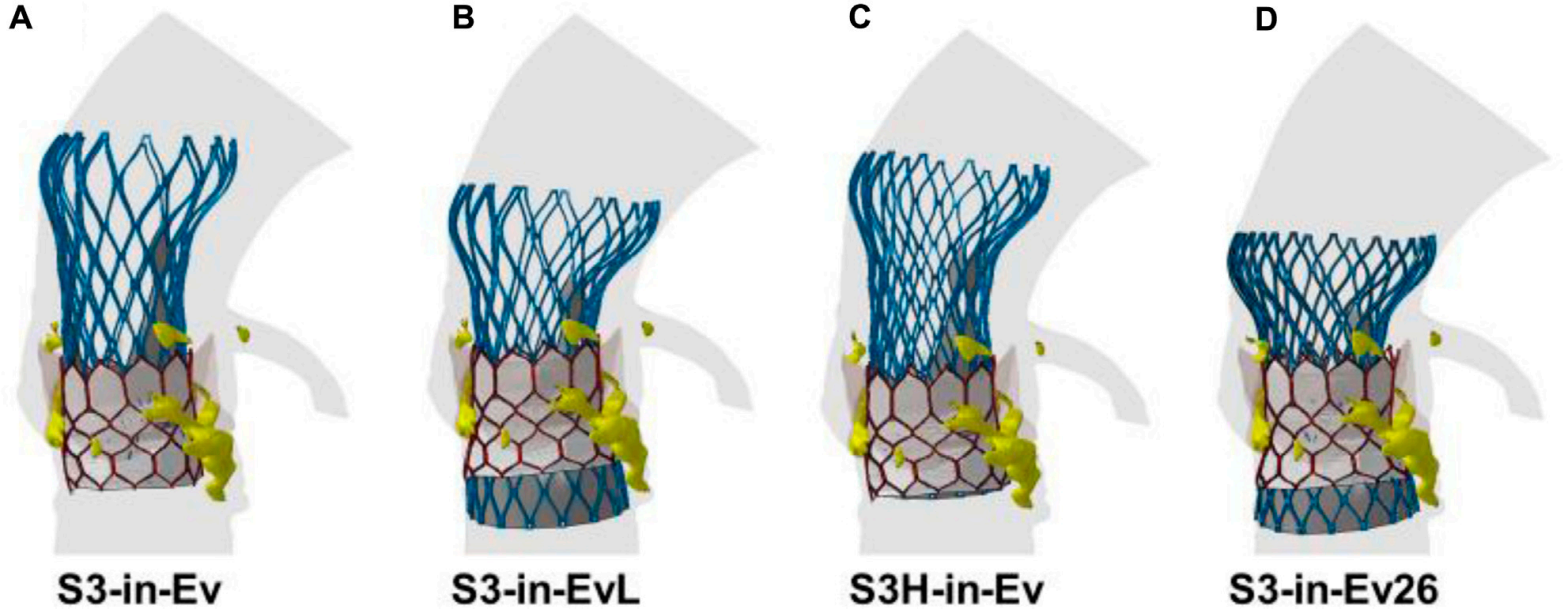
Deformed shapes of TAVR-in-TAVR for **(A)** baseline model, **(B)** low Evolut Pro implantation depth, **(C)** high SAPIEN 3 Ultra implantation depth, **(D)** Evolut Pro undersize.

A qualitative comparison was performed between FEA and imaging of TAVR-in-TAVR for the reference S3-in-EV model, which mimicked the implantation depth performed by the Heart Team (Figure 3). The expansion index was used to quantify the level of agreement between FEA and CT images (Figure 4). The predicted estimations of the expansion index showed good agreement with CT-based measurements at the device inflow, mid-level, and device outflow. After TAVR-in-TAVR, the expansion index decreased by 40% in the inflow region due to the severe calcific plaques near the left ventricular outflow tract of the patient. Furthermore, a good agreement was found for the measurement of VTC distance between simulations and CT images. Deformed shapes resulting from TAVR-in-TAVR simulations highlighted different device shape configurations, which strongly depended on the device size or the extension of the implantation depth (Figure 5).

**FIGURE 6:**
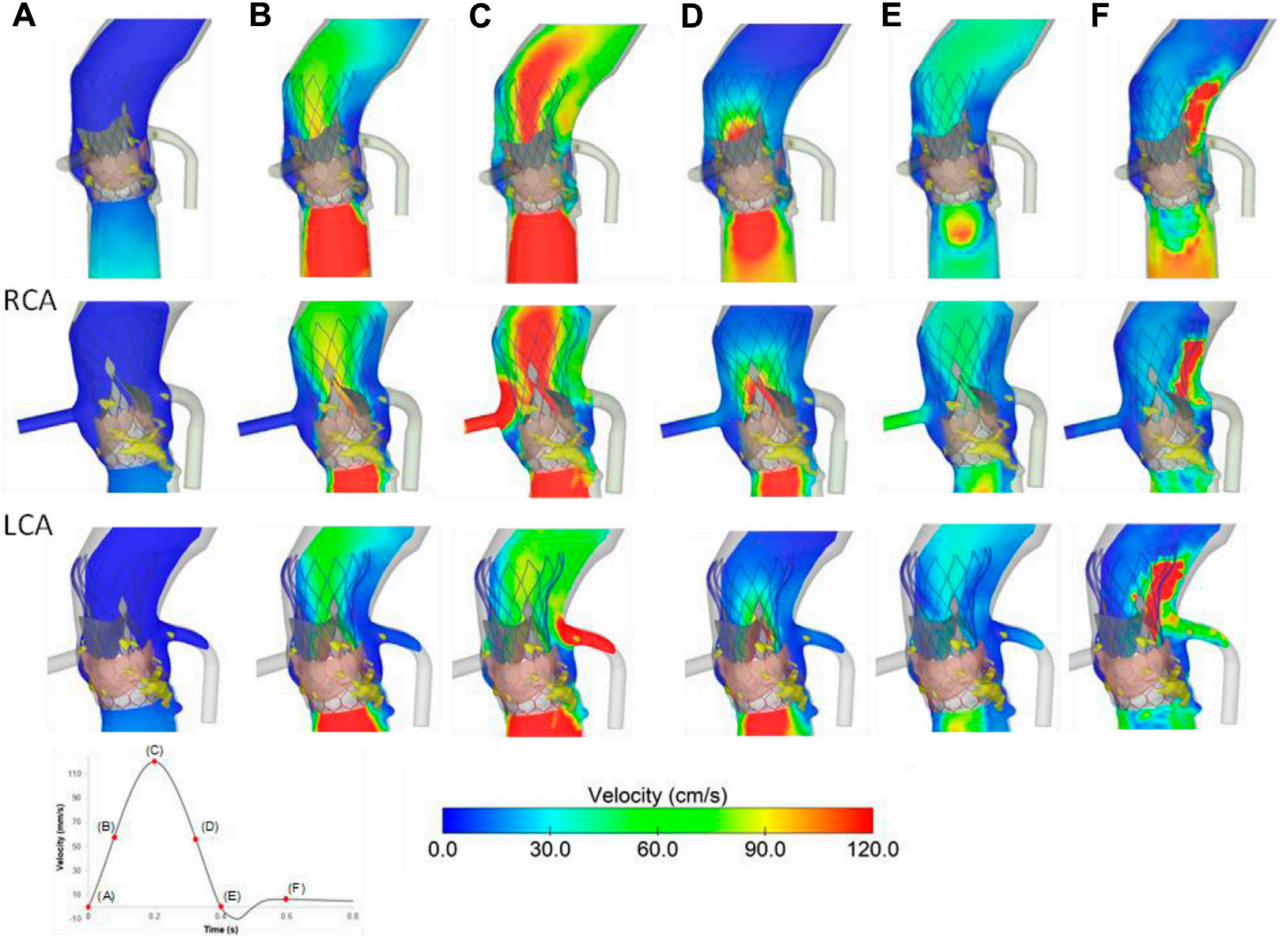
Flow velocities for the baseline model at different cardiac phases **(A–F)**; velocities in RCA (mid row) and LCA (bottom row) are shown.

**FIGURE 7:**
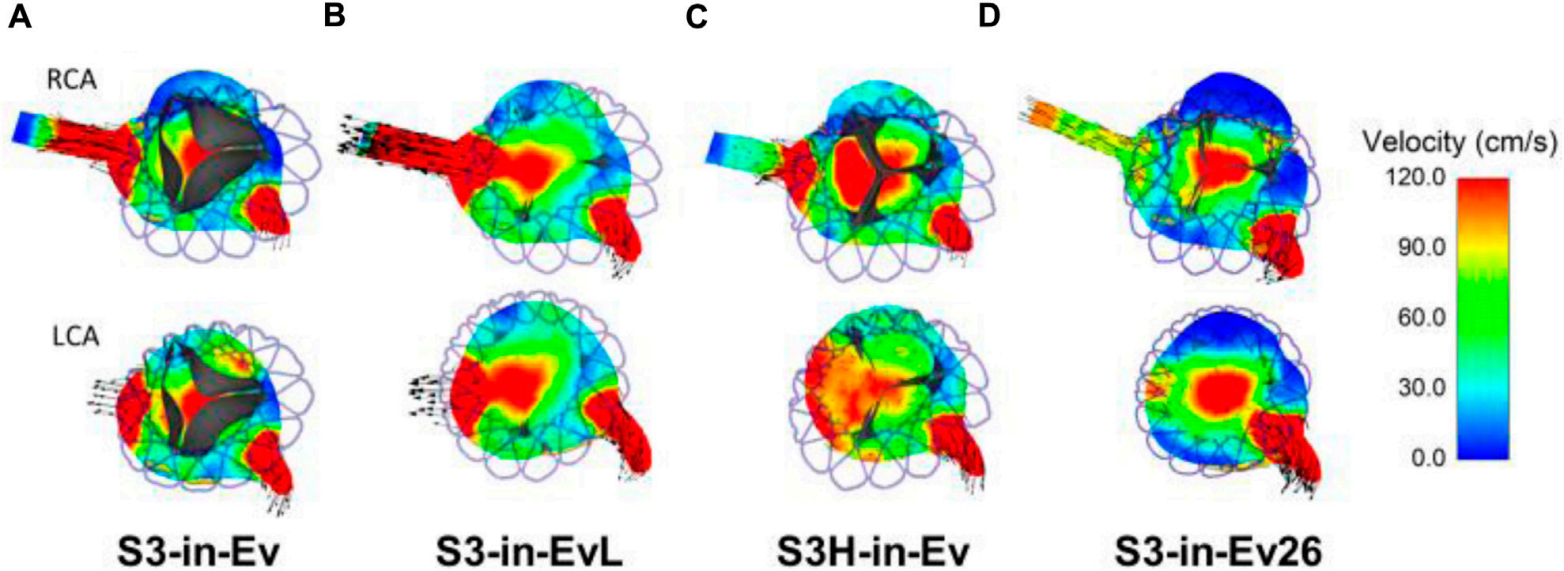
Flow velocities in the axial view for various TAVR-in-TAVR; **(A)** baseline model, **(B)** low Evolut Pro implantation depth **(C)** high SAPIEN 3 Ultra implantation depth, **(D)** Evolut Pro undersize at early diastole just before valve closure.

The flow velocities over the cardiac beat were analyzed to investigate the flow circulating in the coronary arteries after TAVR-in-TAVR (Figure 6). During peak systole, a central flow jet from the opened device valve leaflets was observed while high flow velocities were noted at the end of the deceleration phase when the valve is almost closed. Mid-diastole showed the phase with the most pronounced coronary flow circulation (see Figure 6E). A region of high flow velocities was observed above the LCA ostium, suggesting that the increased flow velocity is likely due to a narrowing region confined by the aortic wall and the Evolut device skirt. The alignment of the leaflet commissures of Evolut Pro with those of the native aortic valve ensured coronary circulation, thereby reducing the effect of the tall device skirt on coronary obstruction. Figure 7 depicts the map of flow velocities at early-diastole (i.e., just before valve closure) for various implantation scenarios. The S3-in-Ev26 model had lower coronary flow velocities than those observed in other models, likely due to undersizing of Evolut PRO. The model with low implantation depth of Evolut PRO in the SAPIEN 3 Ultra device (i.e., S3-in-EvL) highlighted high coronary flow velocities, while the model with high implantation depth for the first device mainly altered the flow velocity of the RCA vessel.

**FIGURE 8:**
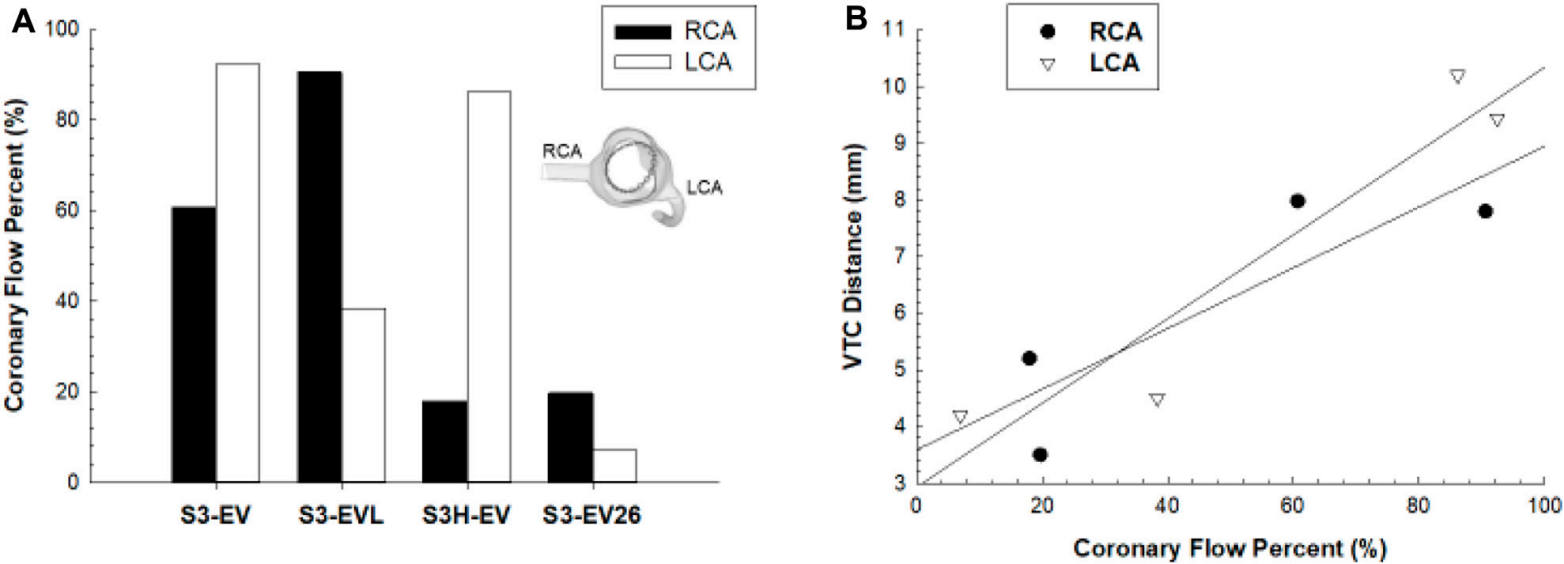
**(A)** Comparison of coronary flow percent between RCA and LCA in different implantation configurations; **(B)** linear regression analysis between coronary flow percent and VCT distance.

Figure 8A illustrates the coronary flow percent changes for different models. A reduction of coronary flows was observed for the S3-in-Ev26 model as compared to the pre-TAVR stage. For the reference configuration (i.e., S3-in-Ev), the LCA flow was reduced to 60% with respect to pre-TAVR model, while the RCA flow remained relatively unchanged. At Pearson correlation, a strong positive linear relationship of coronary flow percent with the VTC distance was observed (see Figure 8B). The VTC distance from the device frame to the LCA ostia was positively correlated with LCA flow (R = 0.86 and *p* = 0.032), while the correlation between VTC distance and RCA coronary flow was stronger (R = 0.93 and *p* = 0.014).

## 4 Discussion

In this study, computational modeling was used to investigate the impact of TAVR-in-TAVR on coronary flow as the placement of the second prosthetic valve in the degenerated device can cause changes in the hemodynamics of the aortic root. The parametric analysis we conducted revealed that variations in the implantation depth or device size can alter the flow circulation compared to pre-TAVR flow analysis. We also discovered that the VTC distance, used as a pre-procedural metric for patient risk stratification, correlates with the amount of flow circulating in the coronary arteries. While this study is limited to this specific patient case, the identification of design and positioning strategies that optimize coronary flow has the potential to enhance the clinical outcomes of TAVR-in-TAVR procedures and mitigate the risk of adverse events.

Coronary artery obstruction is four times more common for valve-in-valve deployment when compared to traditional TAVR, as the leaflets of the degenerated THV become tilted vertically by the second device, creating a cylindrical covered stent frame (McElhinney et al., 2016). Thus, TAVR-in-TAVR may be unfeasible in a significant proportion of patients, especially those treated with a tall frame like the Evolut Pro during the initial procedure. To minimize the likelihood of coronary obstruction, safety imaging guidelines based on geometric factors of the patient’s aortic root were introduced (Forrestal et al., 2020; Nai Fovino et al., 2020). For THV in failed aortic bioprostheses, a short VTC distance evaluated by CT (<4 mm) was found as a predictor of complications in TAVR-in-TAVR (Ribeiro et al., 2018). The permanent displacement of the first implanted THV may lead to coronary obstruction with a mechanism similar to transcatheter mitral valve replacement, where the device displaces permanently the anterior mitral valve leaflets towards the intraventricular septum (Pasta et al., 2020b; Pasta et al., 2022). This leads to a narrowing of left ventricular outflow tract, which can ultimately lead to hemodynamic impairment. For this reason, Khan et al. (2019) have proposed a percutaneous treatment using an electrosurgical catheter to lacerate the pericardial tissue of the first device and thus restore the coronary access. This solution was indeed elaborated on the basis of the LAMPOON procedure (Babaliaros et al., 2017), which lacerates the anterior mitral valve leaflet to prevent outflow obstruction in transcatheter mitral valve replacement. During diastole, coronary perfusion is reliant on the pressure drop between the myocardial tissue and the coronary ostia of the aortic root wall. The flow field near the device closing valve leaflets and coronary resistance have been shown to impact this pressure difference, as demonstrated by Wald et al. (2018).

Several computational studies have examined the effects of various procedural factors on TAVR configuration, including the implantation depth and tilt angle (Finotello et al., 2017), the utilize of self-expanding devices (Ghosh et al., 2020; Pasta et al., 2021) or balloon-expandable devices (Bianchi et al., 2019). Additionally, simulations were used to examine the efficacy and safety of TAVR in patients at high risk or with bicuspid aortic valves (Brouwer et al., 2018; Pasta et al., 2020a). However, there remains a dearth of computational studies focused on evaluating coronary flow in TAVR procedures. Heitkemper et al. (2020) highlighted that neither coronary ostium height nor sinus of Valsalva diameter are reliable predictors of coronary obstruction in high-risk patients, specifically those with a coronary ostium height of less than 14 mm and/or sinus diameter of less than 30 mm. To the best of our knowledge, this is the first computational study investigating coronary flow in TAVR-in-TAVR. Our findings indicate that there is a positive correlation between VTC distance and coronary flow (i.e., the larger the VTC distance, the higher the coronary flow). This relationship is reasonable since patient geometry was not altered in this study, and thus the impact of sinus anatomy on coronary obstruction was not considered. Using particle image velocimetry, the mechanistic link between valve thrombosis and neo-sinus hemodynamic, as the flow confined by native sinus and THV, was demonstrated to vary with the deployment strategy and device size (Midha et al., 2017). Recently, an experimental analysis was conducted to explore the hemodynamics of TAVR-in-TAVR by combining the SAPIEN 3 and Evolut PRO devices to replicate several TAVR-in-TAVR scenarios (Hatoum et al., 2021). They demonstrated that the choice of the optimal configuration of the second THV strongly depends on the first device deployment since the flow turbulence is generally higher than traditional TAVR and is influenced by the interaction between the patient’s anatomy and the device. In our study, the baseline configuration had the distal ends of SAPIEN 3 Ultra at the level of LCA ostia while the coronary orifices are beyond the Evolut PRO skirt and the commissural posts to generate a coaxial intubation. The Evolut PRO valve leaflets faces the coronary arteries for both the reference configuration and low implantation configuration, and this may explain the modest flow-related differences among these models. Differently, the hemodynamics of the coronary arteries were markedly affected by a high implantation depth of SAPIEN 3 Ultra or an undersized Evolut PRO.

### 4.1 Limitations

To avoid numerical issues due to large displacements and complex contact conditions, the SAPIEN 3 Ultra valve leaflets were not simulated in this study. This may have altered the resulting coronary flow estimates; although, the covering of whole stent frame with a surface has most likely resulted in the analysis of the worst case scenario. Most importantly, the SPH method is limited by the particle size and not any verification on the mesh sensitivity was carried out in consideration of the small caliber of coronary vessels. Furthermore, the distal ends of each outlet were closed off with a surface to prevent particle dispersion outside the solid domain. This could have affected the blood flow conditions at the boundary, despite the outlets being adequately extended. This can impact the blood flow conditions at the boundary, though the outlets were sufficiently extended. A Neo-Hookean material model was used for the aortic root model, despite the fact that the soft tissue is hyperelastic and anisotropic materials. A Neo-Hookean material model was used for the aortic root model and the device valve leaflets, despite the fact that the soft tissue is hyperelastic and anisotropic materials. However, imaging data of patient undergoing TAVI suggests stiffening of the aortic wall with very low circumferential strains due to patient’s advanced age. Our assumption aligns with the findings of Bosi et al. (2020), who validated the utilize of linear elastic material properties for the aortic wall and valve leaflets on a cohort of twenty patients underwent TAVI using echocardiographic imaging. The fidelity of the segmentation process and reconstruction of stenotic valve leaflets was not examined in this study. Moreover, the pre-stress of segmented aortic root geometries was not considered due to advanced patient age and resultant vessel stiffening. Subsequent investigations should prioritize the selection of the segmentation procedure and conduct an analysis to assess the accuracy of the geometric models, as this can significantly influence the estimations of both structural and hemodynamic factors here investigated. Future studies should increase the sample size for Pearson’s correlation and consider other TAVR-in-TAVR configurations not considered here.

## 5 Conclusion

This study revealed important insights into the deployment of THVs when the first device experiences structural valve failure. The risk of device obstruction on the resulting coronary flow after TAVR-in-TAVR depends on both the original implant and the device to be implanted. By using computational models to simulate blood flow patterns and assess the impact of different deployment strategies on coronary flow, clinicians can better predict and manage the risk of coronary obstruction during TAVR-in-TAVR procedures. Future studies should continue to investigate the impact of different factors such as patient anatomy, implantation depth, and device size on coronary flow, especially in TAVR-in-TAVR.

## Data Availability

The raw data supporting the conclusion of this article will be made available by the authors, without undue reservation.
